# Supplementary material for: Modeling glioblastoma heterogeneity as a dynamic network of cell states
Source: Mol Syst Biol. 2021 Sep 16;17(9):e10105. doi: 10.15252/msb.202010105 (PMC8444284; doi:10.15252/msb.202010105)
Supplement: Supplementary file 6 — Source Data for Figure 5 [file MSB-17-e10105-s004.zip › Figure5A_sourcedata/GSEA_3071/hallmarks_stateVI.GseaPreranked.1623742557031/HALLMARK_HYPOXIA.html]

Details for gene set HALLMARK\_HYPOXIA[GSEA]

|  || Dataset | state63071 |
| Phenotype | NoPhenotypeAvailable |
| Upregulated in class | na\_pos |
| GeneSet | HALLMARK\_HYPOXIA |
| Enrichment Score (ES) | 0.33804464 |
| Normalized Enrichment Score (NES) | 2.36993 |
| Nominal p-value | 0.0 |
| FDR q-value | 0.0 |
| FWER p-Value | 0.0 |
Table: GSEA Results Summary

  

Fig 1: Enrichment plot: HALLMARK\_HYPOXIA      
 Profile of the Running ES Score & Positions of GeneSet Members on the Rank Ordered List

  

| PROBE | GENE SYMBOL | GENE\_TITLE | RANK IN GENE LIST | RANK METRIC SCORE | RUNNING ES | CORE ENRICHMENT || 1 | SERPINE1 |  |  | 0 | 1.673 | 0.0336 | Yes |
| 2 | TGM2 |  |  | 3 | 1.367 | 0.0606 | Yes |
| 3 | CITED2 |  |  | 5 | 1.232 | 0.0851 | Yes |
| 4 | MT1E |  |  | 10 | 1.128 | 0.1066 | Yes |
| 5 | CAV1 |  |  | 13 | 1.087 | 0.1280 | Yes |
| 6 | DDIT3 |  |  | 14 | 1.086 | 0.1498 | Yes |
| 7 | HOXB9 |  |  | 21 | 1.032 | 0.1689 | Yes |
| 8 | F3 |  |  | 26 | 0.988 | 0.1876 | Yes |
| 9 | LOX |  |  | 29 | 0.968 | 0.2065 | Yes |
| 10 | PLAUR |  |  | 34 | 0.936 | 0.2243 | Yes |
| 11 | CAVIN1 |  |  | 44 | 0.888 | 0.2396 | Yes |
| 12 | MT2A |  |  | 58 | 0.859 | 0.2532 | Yes |
| 13 | ANXA2 |  |  | 79 | 0.783 | 0.2633 | Yes |
| 14 | AKAP12 |  |  | 92 | 0.743 | 0.2749 | Yes |
| 15 | PPP1R15A |  |  | 98 | 0.738 | 0.2884 | Yes |
| 16 | DUSP1 |  |  | 136 | 0.685 | 0.2918 | Yes |
| 17 | ADORA2B |  |  | 192 | 0.608 | 0.2885 | Yes |
| 18 | LDHA |  |  | 197 | 0.604 | 0.2996 | Yes |
| 19 | ENO1 |  |  | 223 | 0.587 | 0.3044 | Yes |
| 20 | MAFF |  |  | 253 | 0.562 | 0.3075 | Yes |
| 21 | MYH9 |  |  | 312 | 0.534 | 0.3020 | Yes |
| 22 | JUN |  |  | 321 | 0.527 | 0.3103 | Yes |
| 23 | GLRX |  |  | 330 | 0.523 | 0.3186 | Yes |
| 24 | NR3C1 |  |  | 395 | 0.492 | 0.3105 | Yes |
| 25 | ACKR3 |  |  | 408 | 0.486 | 0.3169 | Yes |
| 26 | HSPA5 |  |  | 414 | 0.484 | 0.3252 | Yes |
| 27 | ATF3 |  |  | 416 | 0.483 | 0.3347 | Yes |
| 28 | TNFAIP3 |  |  | 445 | 0.473 | 0.3363 | Yes |
| 29 | PFKP |  |  | 473 | 0.463 | 0.3380 | Yes |
| 30 | ERRFI1 |  |  | 526 | 0.449 | 0.3325 | No |
| 31 | PIM1 |  |  | 582 | 0.430 | 0.3257 | No |
| 32 | EGFR |  |  | 669 | 0.407 | 0.3097 | No |
| 33 | FOSL2 |  |  | 717 | 0.393 | 0.3044 | No |
| 34 | KLF6 |  |  | 854 | 0.368 | 0.2736 | No |
| 35 | NFIL3 |  |  | 956 | 0.352 | 0.2523 | No |
| 36 | TPI1 |  |  | 972 | 0.350 | 0.2551 | No |
| 37 | SDC4 |  |  | 1013 | 0.343 | 0.2508 | No |
| 38 | PGK1 |  |  | 1099 | 0.331 | 0.2336 | No |
| 39 | ADM |  |  | 1185 | 0.318 | 0.2161 | No |
| 40 | NOCT |  |  | 1206 | 0.315 | 0.2168 | No |
| 41 | JMJD6 |  |  | 1580 | 0.271 | 0.1175 | No |
| 42 | GCNT2 |  |  | 1598 | 0.269 | 0.1181 | No |
| 43 | SLC2A1 |  |  | 1606 | 0.268 | 0.1215 | No |
| 44 | PDK1 |  |  | 1609 | 0.268 | 0.1264 | No |
| 45 | MIF |  |  | 1618 | 0.267 | 0.1295 | No |
| 46 | ISG20 |  |  | 1756 | 0.255 | 0.0962 | No |
| 47 | SDC3 |  |  | 1818 | -0.252 | 0.0841 | No |
| 48 | GPC1 |  |  | 1876 | -0.261 | 0.0733 | No |
| 49 | CASP6 |  |  | 1996 | -0.278 | 0.0455 | No |
| 50 | HK1 |  |  | 2053 | -0.287 | 0.0355 | No |
| 51 | VEGFA |  |  | 2104 | -0.298 | 0.0275 | No |
| 52 | VHL |  |  | 2288 | -0.330 | -0.0173 | No |
| 53 | CHST3 |  |  | 2337 | -0.340 | -0.0239 | No |
| 54 | KLHL24 |  |  | 2407 | -0.355 | -0.0362 | No |
| 55 | SAP30 |  |  | 2413 | -0.356 | -0.0304 | No |
| 56 | ZNF292 |  |  | 2426 | -0.360 | -0.0266 | No |
| 57 | PHKG1 |  |  | 2530 | -0.384 | -0.0478 | No |
| 58 | BGN |  |  | 2551 | -0.388 | -0.0456 | No |
| 59 | KIF5A |  |  | 2650 | -0.412 | -0.0648 | No |
| 60 | TGFBI |  |  | 2679 | -0.419 | -0.0643 | No |
| 61 | PRKCA |  |  | 2847 | -0.474 | -0.1016 | No |
| 62 | CCNG2 |  |  | 2927 | -0.504 | -0.1137 | No |
| 63 | DCN |  |  | 3027 | -0.554 | -0.1304 | No |
| 64 | FOXO3 |  |  | 3053 | -0.564 | -0.1260 | No |
| 65 | PNRC1 |  |  | 3055 | -0.566 | -0.1149 | No |
| 66 | SDC2 |  |  | 3080 | -0.578 | -0.1101 | No |
| 67 | WSB1 |  |  | 3130 | -0.608 | -0.1116 | No |
| 68 | PAM |  |  | 3238 | -0.708 | -0.1274 | No |
| 69 | CXCR4 |  |  | 3302 | -0.759 | -0.1298 | No |
| 70 | NCAN |  |  | 3329 | -0.794 | -0.1212 | No |
| 71 | HEXA |  |  | 3370 | -0.840 | -0.1155 | No |
| 72 | STC1 |  |  | 3393 | -0.880 | -0.1040 | No |
| 73 | CDKN1B |  |  | 3426 | -0.951 | -0.0939 | No |
| 74 | NEDD4L |  |  | 3469 | -1.045 | -0.0846 | No |
| 75 | FOS |  |  | 3476 | -1.056 | -0.0651 | No |
| 76 | CSRP2 |  |  | 3484 | -1.081 | -0.0453 | No |
| 77 | PDGFB |  |  | 3506 | -1.131 | -0.0284 | No |
| 78 | MAP3K1 |  |  | 3584 | -1.605 | -0.0178 | No |
| 79 | IGFBP3 |  |  | 3588 | -1.623 | 0.0140 | No |
Table: GSEA details [plain text format]

  

Fig 2: HALLMARK\_HYPOXIA: Random ES distribution      
 Gene set null distribution of ES for **HALLMARK\_HYPOXIA**
